# Diarrhetic Shellfish Toxins and Other Lipophilic Toxins of Human Health Concern in Washington State

**DOI:** 10.3390/md11061815

**Published:** 2013-05-28

**Authors:** Vera L. Trainer, Leslie Moore, Brian D. Bill, Nicolaus G. Adams, Neil Harrington, Jerry Borchert, Denis A. M. da Silva, Bich-Thuy L. Eberhart

**Affiliations:** 1 Marine Biotoxins Program, Environmental Conservation Division, Northwest Fisheries Science Center, National Marine Fisheries Service, National Oceanic and Atmospheric Administration, 2725 Montlake Blvd. E, Seattle, WA 98112, USA; E-Mails: leslie.moore@noaa.gov (L.M.); brian.d.bill@noaa.gov (B.D.B.); nicolaus.adams@noaa.gov (N.G.A.); denis.dasilva@noaa.gov (D.A.M.S.); bich-thuy.le.eberhart@noaa.gov (B.-T.L.E.); 2 Jamestown S’Klallam Tribe, 1033 Old Blyn Highway, Sequim, WA 98392, USA; E-Mail: nharrington@jamestowntribe.org; 3 Office of Shellfish and Water Protection, Washington State Department of Health, 111 Israel Rd SE, Tumwater, WA 98504, USA; E-Mail: jerry.borchert@doh.wa.gov

**Keywords:** diarrhetic shellfish toxins, diarrhetic shellfish poisoning, DSP, *Dinophysis*, harmful algal bloom, SoundToxins, ORHAB

## Abstract

The illness of three people in 2011 after their ingestion of mussels collected from Sequim Bay State Park, Washington State, USA, demonstrated the need to monitor diarrhetic shellfish toxins (DSTs) in Washington State for the protection of human health. Following these cases of diarrhetic shellfish poisoning, monitoring for DSTs in Washington State became formalized in 2012, guided by routine monitoring of *Dinophysis* species by the SoundToxins program in Puget Sound and the Olympic Region Harmful Algal Bloom (ORHAB) partnership on the outer Washington State coast. Here we show that the DSTs at concentrations above the guidance level of 16 μg okadaic acid (OA) + dinophysistoxins (DTXs)/100 g shellfish tissue were widespread in sentinel mussels throughout Puget Sound in summer 2012 and included harvest closures of California mussel, varnish clam, manila clam and Pacific oyster. Concentrations of toxins in Pacific oyster and manila clam were often at least half those measured in blue mussels at the same site. The primary toxin isomer in shellfish and plankton samples was dinophysistoxin-1 (DTX-1) with *D. acuminata* as the primary *Dinophysis* species. Other lipophilic toxins in shellfish were pectenotoxin-2 (PTX-2) and yessotoxin (YTX) with azaspiracid-2 (AZA-2) also measured in phytoplankton samples. Okadaic acid, azaspiracid-1 (AZA-1) and azaspiracid-3 (AZA-3) were all below the levels of detection by liquid chromatography tandem mass spectrometry (LC-MS/MS). A shellfish closure at Ruby Beach, Washington, was the first ever noted on the Washington State Pacific coast due to DSTs. The greater than average Fraser River flow during the summers of 2011 and 2012 may have provided an environment conducive to dinoflagellates and played a role in the prevalence of toxigenic *Dinophysis* in Puget Sound.

## 1. Introduction

Diarrhetic shellfish poisoning (DSP) is a syndrome in humans caused by the ingestion of shellfish contaminated by toxins produced by dinoflagellates in the genera *Dinophysi*s and *Prorocentrum* [1,2,3]. DSP symptoms include diarrhea, nausea, vomiting, and abdominal pain starting 30 min to a few hours after ingestion of the toxic shellfish, with complete recovery within three days [4]. Tumor-promoting, mutagenic and immunosuppressive effects shown in animals to be associated with diarrhetic shellfish toxins (DSTs) including okadaic acid (OA) and the dinophysistoxins (DTXs) have not yet been quantified in humans [5]. However there is speculation that chronic exposure may increase the risk of gastrointestinal cancers [6,7,8]. The earliest clinical reports of DSP were from the Netherlands in 1961, but it was not until 1976 that the DSTs were identified in Japan where they caused major problems for the scallop fishery [1,9,10]. Between 1976 and 1982, some 1300 DSP cases were reported in Japan, in 1981 more than 5000 cases were reported in Spain, and in 1983 some 3300 cases were reported in France. In 1984, DSP caused a shutdown of the mussel industry for almost a year in Sweden. The known global distribution of DSTs includes Japan, Europe, Asia, Chile, Canada, Tasmania, New Zealand [11], with recent confirmation in U.S. shellfish [12,13]. The first clinical report of DSP in the U.S. with coincident high concentrations of DSTs in shellfish occurred in 2011 in Washington State.

Three DSP illnesses were reported on June 29, 2011 in the US Pacific Northwest from the consumption of mussels collected from a pier at Sequim Bay State Park. Blue mussels collected within a few days of the illnesses were found by LC-MS/MS analysis to contain levels of DSTs 2–10 times the action level, resulting in closure to recreational and commercial harvest of shellfish and product recalls. Nine mussel samples exhibited toxin levels above the regulatory action threshold, ranging from 37.6 to 160.3 μg/100 g shellfish tissue. Coincidentally, roughly 60 DSP illnesses occurred in July–August, 2011 on Salt Spring Island, British Columbia, traced to ingestion of Pacific coast mussels, representing the first reports of DSP in western Canada [14]. Almost 14,000 kg of product was recalled. Although the presence of *Dinophysis* in Pacific Northwest coastal waters dates back many years [15], this was the first time illnesses were reported in conjunction with DST levels deemed hazardous to human health.

The lipophilic toxins in shellfish can be divided into four groups of toxins with different chemical structures and biological effects: OA and its derivatives, the DTXs; the pectenotoxins (PTXs); the yessotoxins (YTXs); and the azaspiracids (AZAs). These toxins can often be found in combination in shellfish. Both OA and the DTXs are acid polyethers that inhibit protein phosphatase [16,17], and are the only toxins of the DSP complex with diarrheagenic effects in mammals [5]. Some of the PTXs are hepatotoxic to mice by intraperitoneal injection, and the YTXs are cardiotoxic to mice [18], but have, to date, not been associated with human poisonings [19]. Neither the YTXs, nor PTX-2 and its shellfish-mediated derivative PTX-2-secoacid, are toxic to mice when administered orally [20,21,22], and their potential threat to human health is currently being debated [19]. These three groups of toxins can now be analyzed with independent analytical methods, which led the European Union (EU) to regulate them separately [23]. The history of misidentifications of the causative toxins and the agents of diarrhetic toxin outbreaks over the past three decades may be attributed to the following: both OA and the PTXs are produced by *Dinophysis*; OA is also produced by benthic dinoflagellates of the genus *Prorocentrum*, but *P. micans*, a species that frequently co-occurs with *D. acuminata*, is not a toxin-producer; the producers of YTXs (*Lingulodinium polyedrum*, *Protoceratium reticulatum*, *Gonyaulax spinifera*) and of AZAs (*Azadinium spinosum*) often co-occur in assemblages of lipophilic toxin-producers; all lipophilic toxins, including OA, DTXs, PTXs, YTXs and AZAs, are co-extracted and give a single cumulative response in conventional mouse bioassays; and, the acute symptoms of DSP are easily confused with gastroenteritis [24]. Furthermore, only recently was the small dinoflagellate *Azadinium spinosum* identified as a source of AZAs [25,26], following years of incorrectly associating the heterotrophic dinoflagellate *Protoperidinium crassipes* with production of the toxin.

DSTs and azaspiracid shellfish toxins have an enormous economic and health impact in Europe but are not currently regulated or monitored in many US coastal states; moreover, very little is known about their distribution and impact on human health. Suspected but not confirmed DSP illnesses have been recorded on the East Coast since 1980, coinciding with the detection of toxin-producing dinoflagellates in shellfish beds [1,3]. In addition to the recent sudden blooms of *Dinophysis* on the Potomac River estuary of Chesapeake Bay [27] and in the Gulf of Mexico in 2008 [12,13], the DSP illnesses in Washington State highlight the urgent need for further investigation of these emerging threats.

**Figure 1:**
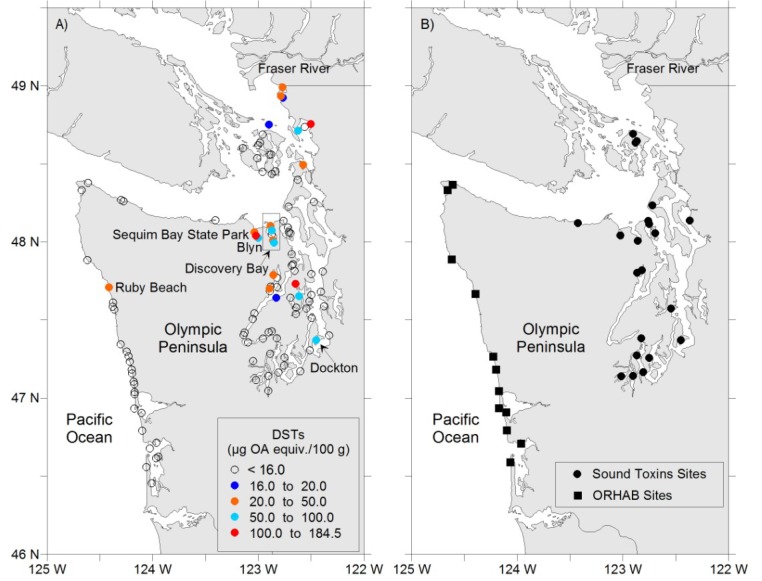
Locations of shellfish monitoring for diarrhetic shellfish toxins (DSTs) in Washington State. Colored symbols represent different concentrations of toxins at sites where regulatory closures occurred at least once from June to October 2012. Open symbols indicate sites where no closures occurred (**A**). SoundToxins and Olympic Region Harmful Algal Bloom (ORHAB) sampling sites that provide weekly phytoplankton abundance data as an early warning of harmful algal events are shown (**B**).

During 2012, shellfish and phytoplankton samples were collected from Washington State coastal waters and analyzed for DSTs. Shellfish analysis was targeted to those areas where *Dinophysis* were observed by the Puget Sound monitoring partnership, SoundToxins, and the outer Washington State coast’s Olympic Region Harmful Algal Bloom (ORHAB) partnership (Figure 1B). Here we document concentrations of *Dinophysis* spp., as well as the detection and confirmation of alert levels of DSTs and other lipophilic marine toxins at multiple sites in Puget Sound and document the first DST-related shellfish closure on the outer Washington State coast.

## 2. Results

Analysis of shellfish samples from May 30 to October 2, 2012 for the regulatory management of DSTs including OA + DTX-1 + DTX-2, quantified in units of OA equivalents (OA equiv) resulted in closures at 20 sites representing 38% of all samples analyzed (Figure 1A; *n* = 350). Shellfish analyzed included blue mussel (*Mytilus edulis*; *n* = 211), varnish clam (Nuttalia obscurata; *n* = 2), California mussel (*Mytilus californianus*; *n* = 27), Pacific oyster (*Crassostrea gigas*; *n* = 45), manila clam (*Venerupis philippinarum*; *n* = 21), littleneck clam (*Leukoma staminea*; *n* = 23), geoduck clam (*Panopea generosa*; *n* = 3), butter clam (*Saxidomus giganteus*; *n* = 4), and razor clam (*Siliqua patula*; *n* = 12). Blue mussel, California mussel, varnish clam, manila clam, and Pacific oyster showed at least one sample above the guidance limit of 16 μg/100 g, whereas the other shellfish did not. Most notable was a value of >190 μg/100 g in August 2012 at a site near the Canadian border. The closure at Ruby Beach, Washington, was the first closure due to DSTs in blue mussel on the Washington State Pacific coast.

**Figure 2:**
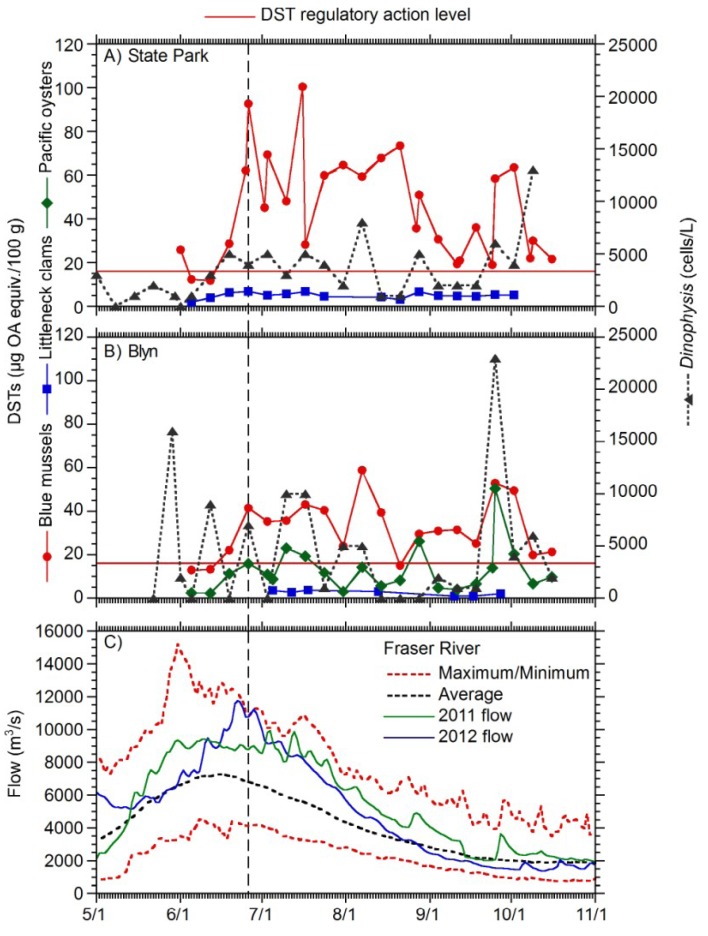
Total DSTs in blue mussels and littleneck clams, and total *Dinophysis* abundance at Sequim Bay State Park (**A**); total DSTs in blue mussels and littleneck clams, Pacific oysters, and total *Dinophysis* abundance at Blyn (**B**). The left *y*-axis shows total DSTs in shellfish (μg okadaic acid (OA) equiv/100 g) and the right y-axis depicts total *Dinophysis* abundance (cells/L). Fraser River average flow, historical minimum and maximum flow (m^3^/s) compared to 2011 and 2012 flow is depicted (**C**). Panels A and B show data from May to November 2012.

In Sequim Bay, weekly phytoplankton and shellfish sampling at both the Sequim Bay State Park pier on the western shore and in the shallower waters of Blyn in the south showed that the first elevated *Dinophysis* counts were observed in early May at the State Park (Figure 2A) and at the end of May when sampling began in Blyn (Figure 2B, see Figure 1A for locations). At that time, LC-MS/MS analysis had not yet been established at the Washington State Department of Health (WDOH). However, the first measurement of DSTs in blue mussel on June 1 at the State Park was 26 μg/100 g, whereas the first sample that exceeded the guidance level in Blyn on June 26 measured 42 μg/100 g in blue mussel. The maximum concentration of DSTs in Sequim Bay was 103 μg/100 g on July 3. Blue mussels showed the highest concentrations of toxins with littleneck clams and Pacific oysters showing maximum concentrations of 7 μg/100 g on July 17 at the State Park and 50 μg/100 g on September 25 at Blyn, respectively (Figure 2). Elevated total *Dinophysis* abundance generally preceded peaks in shellfish toxicity and was higher at Blyn, reaching a maximum abundance of 23,000 cells/L on September 26. The May/June elevated shellfish toxicity correlated with above average flows of the Fraser River during 2012 (Figure 2C and dotted vertical line in Figure 2).

**Figure 3:**
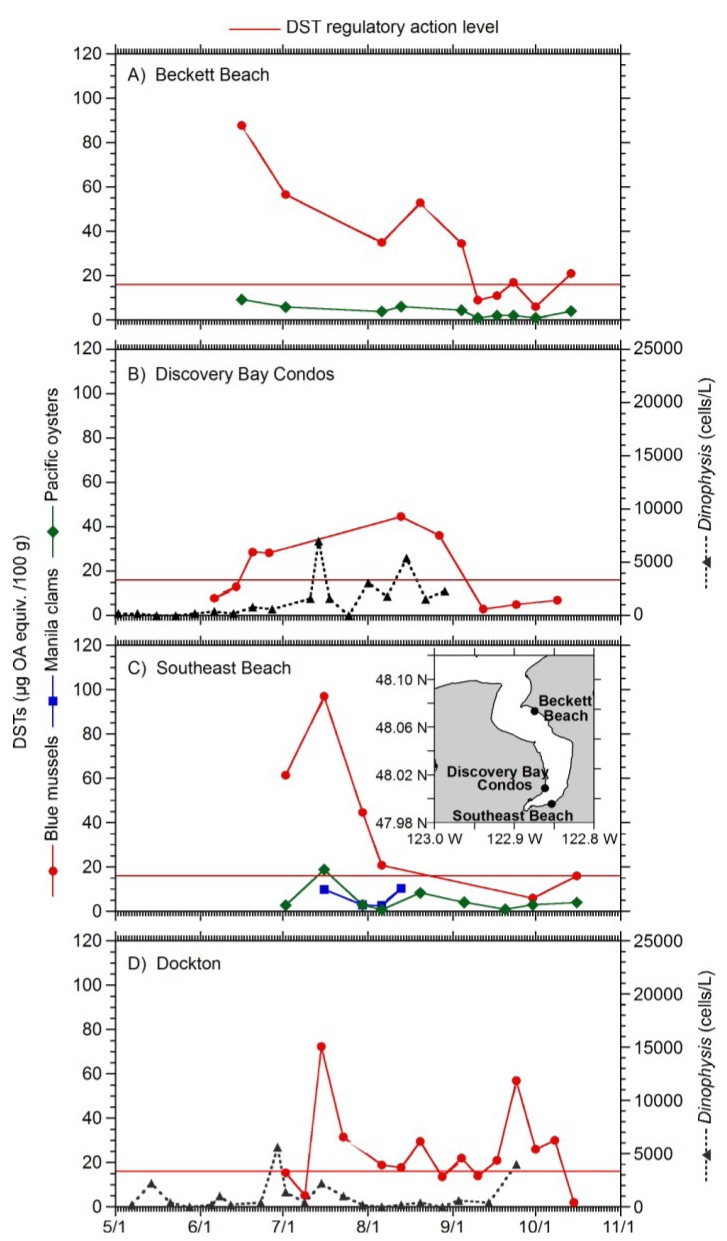
Total DSTs in blue mussels and Pacific oysters at Beckett Beach (**A**); blue mussels at Discovery Bay Condos (**B**); and blue mussels, manila clam, Pacific oyster at Southeast Beach (**C**). Discovery Bay (Figure 1A) monitoring locations are shown (**C**, inset). Total DSTs in blue mussels at Dockton (Figure 1A; **D**). The left *y*-axis depicts total DSTs in shellfish (μg OA equiv/100 g) and *Dinophysis* abundance is shown in the right *y*-axis (cells/L).

The high concentrations of DSTs in Sequim Bay shellfish in early June 2012 motivated the WDOH to expand its analysis of shellfish to other areas of Puget Sound. In Discovery Bay, SoundToxins monitoring of *Dinophysis* abundance at the Discovery Bay Condos did not provide an early warning of shellfish toxicity above the guidance level at any of the sites that were monitored in Discovery Bay (Figure 3). In contrast, at Dockton in Quartermaster Harbor, an increase in *Dinophysis* abundance to 5000 cells/L was observed on June 28, providing a warning two weeks prior to the high toxicity in blue mussels of 72 μg/100 g measured on July 15. However, a *Dinophysis* increase to 4000 cells/L coincided with the second peak in shellfish toxicity in mid-September (Figure 3D). An early warning was missed in this instance, likely because a 10-day interval passed between cell sampling events instead of the usual 1-week period.

**Figure 4:**
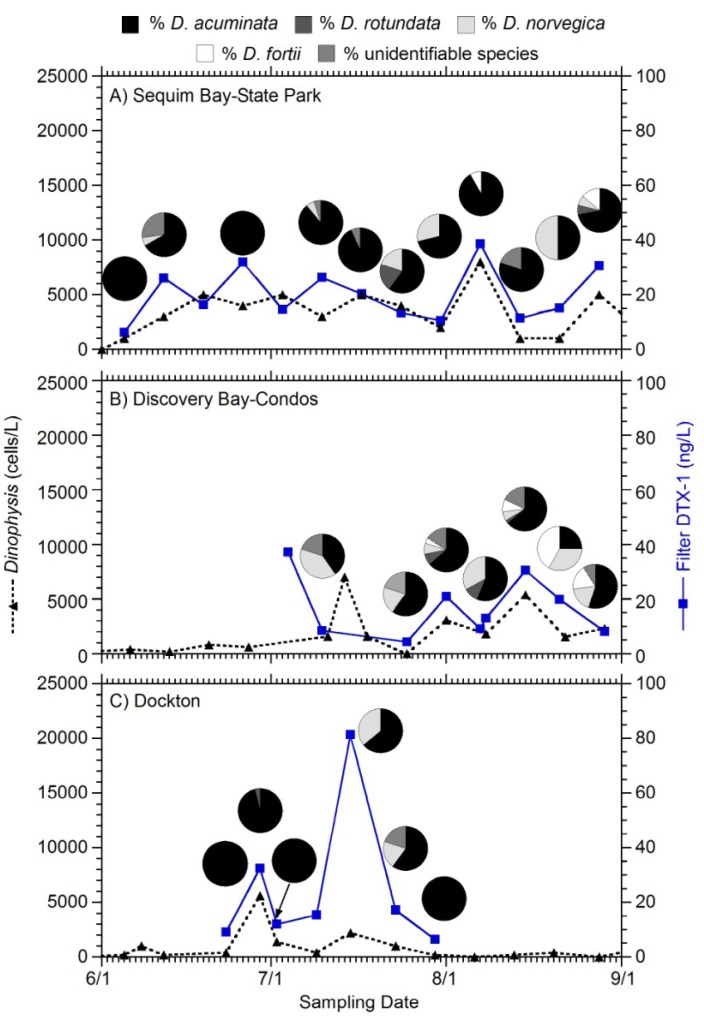
Total *Dinophysis* abundance (cells/L, left *y*-axis) and total DTX-1 (ng/L, right *y*-axis) in filtered plankton samples at Sequim Bay State Park (**A**), Discovery Bay Condos (**B**) and Dockton, Quartermaster Harbor (**C**) during June–September 2012. The pie charts indicate the percentage of each *Dinophysis* species detected on most dates including *D. acuminata*, *D. rotundata*, *D. norvegica*, *D. fortii*, and an unidentifiable species. The location of sampling sites in Discovery Bay (Figure 3C inset) and Dockton (Figure 1A) are shown.

**Figure 5:**
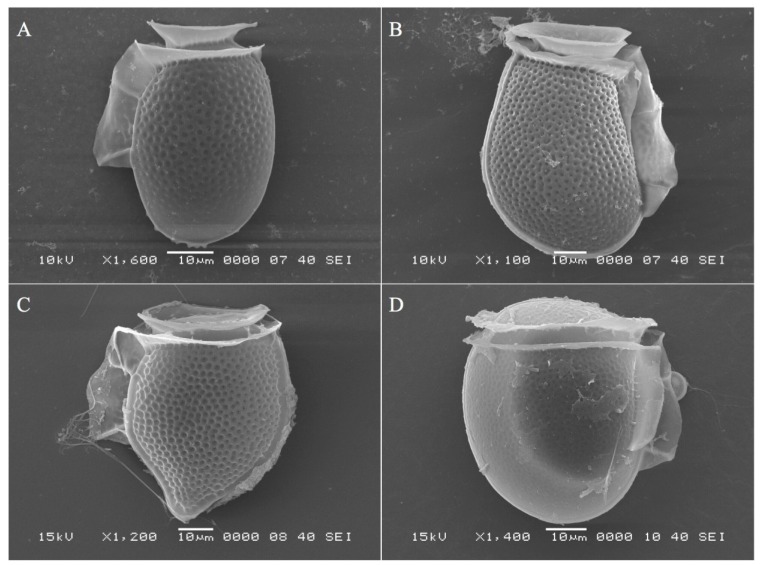
Scanning electron microscope images of selected *Dinophysis* species found in WA State including *D. acuminata* (**A**), *D. fortii* (**B**), *D. norvegica* (**C**), and *D. rotundata* (**D**).

Toxin analysis of filtered plankton samples together with determination of *Dinophysis* species abundance by light microscopy indicate which species contributed to shellfish toxicity in Puget Sound. The majority of samples showed that *D. acuminata* was the main species observed when particulate toxin in filters was measured (Figure 4) with some notable exceptions. For example, on July 15 at Dockton, approximately 20% of the *Dinophysis* were composed of *D. norvegica* (Figure 4C) while total *Dinophysis* abundance was low (2000 cells/L) but particulate toxin concentration was high (72 μg/100 g). Other species such as *D. rotundata* and *D. fortii* were also found in smaller numbers, however it is not possible to determine whether these species contributed to shellfish toxicity in the mixed species samples. Species identities were confirmed by scanning electron microscopy (SEM; Figure 5).

The LC-MS/MS analyses show that DTX-1 was the primary toxin isomer in all shellfish species collected from Sequim Bay (Table 1). Levels of OA were almost negligible in Washington State’s shellfish. The toxin profile was very similar in oysters, clams and mussels. Mussels contained the highest toxin concentrations while oysters and clams generally contained less than half the amount. The relative amount of the acyl ester form in shellfish, termed DTX-3, ranged from 0% to 53% of the total OA equiv in blue mussel (but most often <50% DTX-3), 56% to 100% in Pacific oyster, and 84% to 100% in littleneck clam (but most often 100% DTX-3). On many sampling dates, YTX and PTX-2 were detected in shellfish and plankton filters and AZA-2 was found in plankton filters on July 21, August 7 and 21. Toxin profiles in Sequim Bay shellfish in 2012 (Table 1) were similar in relative proportion to those measured elsewhere in Puget Sound. However, blue mussels from the Pacific coast, including Ruby Beach, also contained small concentrations (<5% of the total DSTs) of OA.

**Table 1:** Lipophilic toxins ^1^ measured in Sequim Bay shellfish and phytoplankton during summer 2012.

Date	Sample	Total OA equiv	DTX-1	DTX-3	YTX	PTX2	AZA-2	*Dinophysis* (cells/L)
6/5/12	Blue Mussel	12.33	5.76	6.57	na ^2^	bd ^3^	bd	1000
	Pacific Oyster	2.55	1.11	1.44	na	bd	bd	
	Littleneck Clam	1.84	0.00	1.84	na	bd	bd	
	Plankton Filter	6.24	3.88	2.36	2.95	5.60	bd	
6/12/12	Blue Mussel	11.80	7.47	4.33	na	bd	bd	3000
	Pacific Oyster	2.35	0.00	2.35	na	bd	bd	
	Littleneck Clam	3.99	0.00	3.99	na	bd	bd	
	Plankton Filter	26.21	19.62	6.59	bd	33.48	bd	
6/19/12	Blue Mussel	28.72	28.72	0.00	na	65.09	bd	5000
	Pacific Oyster	11.31	4.10	7.21	na	59.59	bd	
	Littleneck Clam	6.26	0.00	6.26	na	17.38	bd	
	Plankton Filter	16.37	12.42	3.95	bd	8.24	bd	
6/26/12	Blue Mussel	92.69	54.52	38.17	na	57.16	bd	4000
	Pacific Oyster	15.91	5.59	10.32	na	34.41	bd	
	Littleneck Clam	6.80	5.06	1.74	na	12.58	bd	
	Plankton Filter	32.05	23.14	8.91	bd	13.05	bd	
7/3/12	Blue Mussel	69.35	48.09	21.26	27.32	3.30	bd	5000
	Pacific Oyster	10.95	2.62	8.33	3.70	bd	bd	
	Littleneck Clam	5.08	0.00	5.08	bd	bd	bd	
	Plankton Filter	14.63	9.91	4.72	bd	12.44	bd	
7/10/12	Blue Mussel	48.12	34.22	13.90	89.70	1.46	bd	3000
	Pacific Oyster	23.10	4.24	18.86	bd	3.96	bd	
	Littleneck Clam	5.66	0.00	5.66	bd	bd	bd	
	Plankton Filter	26.41	20.58	5.83	bd	20.82	bd	
7/17/12	Blue Mussel	28.29	13.64	14.65	22.78	bd	bd	5000
	Pacific Oyster	19.38	2.94	16.44	1.78	2.06	bd	
	Littleneck Clam	6.77	1.02	5.75	bd	1.33	bd	
	Plankton Filter	20.36	15.48	4.88	bd	13.52	bd	
7/24/12	Blue Mussel	59.90	44.38	15.52	58.48	1.57	bd	4000
	Pacific Oyster	11.71	2.37	9.34	bd	1.33	bd	
	Littleneck Clam	4.51	0.00	4.51	bd	1.82	bd	
	Plankton Filter	13.35	8.78	4.57	3.00	8.55	bd	
7/31/12	Blue Mussel	64.68	40.60	24.08	33.78	bd	bd	2000
	Pacific Oyster	3.10	1.25	1.85	1.10	bd	bd	
	Littleneck Clam	7.79	1.28	6.51	bd	1.00	bd	
	Plankton Filter	10.58	7.88	2.70	bd	5.91	0.40	
8/7/12	Blue Mussel	59.30	36.28	23.02	30.93	2.83	bd	8000
	Pacific Oyster	14.27	3.65	10.62	1.51	1.70	bd	
	Littleneck Clam	3.99	0.00	3.99	bd	bd	bd	
	Plankton Filter	38.67	29.74	8.93	bd	33.72	0.46	
8/14/12	Blue Mussel	66.61	42.76	23.85	30.20	bd	bd	1000
	Pacific Oyster	5.67	1.96	3.71	2.88	bd	bd	
	Littleneck Clam	4.21	1.10	3.11	1.43	bd	bd	
	Plankton Filter	11.42	10.13	1.29	bd	8.55	bd	
8/21/12	Blue Mussel	73.48	65.98	7.50	37.26	bd	bd	1000
	Pacific Oyster	8.17	2.77	5.40	3.55	2.77	bd	
	Littleneck Clam	3.19	0.00	3.19	1.62	bd	bd	
	Plankton Filter	15.15	10.31	4.84	3.10	18.65	0.40	
8/28/12	Blue Mussel	51.00	42.52	8.48	34.72	2.51	bd	5000
	Pacific Oyster	26.20	5.59	20.61	4.88	3.49	bd	
	Littleneck Clam	6.69	1.69	5.00	bd	1.85	bd	
	Plankton Filter	30.66	22.62	8.04	6.79	38.36	bd	

^1^ OA, DTX-2, AZA-1, AZA-3 were all below the level of detection. Units are μg/100 g for all shellfish and ng/L for plankton filters. ^2^ na = not analyzed. ^3^ bd = below the level of detection.

## 3. Discussion

### 3.1. Dinophysis Monitoring

The production of DSTs has been confirmed in several *Dinophysis* species, including *D. fortii*, *D. acuminata*, *D. acuta*, *D. norvegica*, *D. mitra*, *D. rotundata*, *D. sacculus*, *D. caudata* and *D. tripos*, and in the benthic dinoflagellates *Prorocentrum lima*, *P. concavum* (or *P.* *maculosum*), *P. micans*, *P. minimum* and *P. redfieldii* [28]. One other *Dinophysis* species, *D. hastata*, is also suspected to produce toxins [11]. In the present study, *D. acuminata* is the most abundant DST producer, with DTX-1 as the major toxin. Filtered plankton samples show the same toxin profiles as shellfish samples and no detectable OA was measured in shellfish, suggesting that *Dinophysis*, and not the benthic dinoflagellate *Prorocentrum*, a known producer of OA [29], is likely the primary source of DSTs in Washington State.

For the mixotrophic *Dinophysis*, the primary source of the toxins has been shown to be the dinoflagellate and not its prey [30,31]. However, for heterotrophic species, such as *D. rotundata*, it is likely that their prey is the source of the toxins [32]. Most *Dinophysis* species are often a rare component of the phytoplankton assemblage, occurring at concentrations of 1–100 cells/L, but the species *D. acuminata*, *D. acuta*, *D. caudata*, *D. fortii*, *D. norvegica*, *D.* *rotundata* and *D. sacculus* are able to reach concentrations >10^3^ cells/L in coastal waters and are responsible for chronic DSP events [24]. Reports of DSTs in shellfish have been associated with densities of *Dinophysis* as low as a few hundred cells/L [9]. The appearance of *Dinophysis*, even at low densities such as 200 cells/L, can cause a toxification of shellfish that is enough to affect humans [33]. In Europe, temporary closures and intensified monitoring are initiated when cell densities of target phytoplankton reach 500–1200 cells/L and operations are closed at a threshold level of 5000 cells/L until such time as toxin levels in shellfish are proven to be safe [34]. In our study, the maximum *Dinophysis* abundance was almost 2.5 × 10^4^ cells/L in Blyn in late September (Figure 2B), demonstrating that higher numbers of cells are observed in Washington State than are typically measured in the EU and elsewhere. Conversely, low abundances of *Dinophysis* are also frequently observed in Washington State, so precautionary closures based on cell abundance will not be useful. However, observations of *Dinophysis* abundance show promise in providing early warning to DSP events, as shown from data collected at Sequim Bay State Park, where samples were taken at 2 m depth with a Niskin bottle (Figure 2A), at Blyn (Figure 2B) where water depths are shallow (<1 m), and at Dockton where samples are taken from the surface consistently on incoming tides (Figure 3D). However, in Discovery Bay, surface whole water and net tow samples did not show elevated concentrations of *Dinophysis* prior to shellfish closures. *Dinophysis* are known to exist in thin layers [35] that can be dispersed through the surface water column mixed zone during events such as summer storms. In the future, the SoundToxins partnership will explore the use of integrated tube samplers [36] for samples collected from docks and piers to allow for capture of *Dinophysis* in such layers.

### 3.2. Environmental Triggers

Although *Dinophysis* spp. have been observed in Pacific Northwest for many years [15], DSP has only recently become a major concern in this region, illustrated by the three illnesses in Puget Sound and >60 illnesses in British Columbia in 2011 [14,37] and the widespread DST-related shellfish closures throughout Puget Sound in 2012. What factors have contributed to this recent trend? It is known that 2011 and 2012 were La Niña years with above average snowpack resulting in massive volumes of freshwater from the Fraser River into the Salish Sea in the late spring and early summer (Figure 2). Dinoflagellates are known to thrive in stratified systems and *Dinophysis* has particular adaptive strategies to cope with freshwater plumes [35]. Decade-long phytoplankton records from Monterey Bay provide evidence that the U.S. west coast may have entered a “dinoflagellate regime shift” [38] suggesting that toxigenic *Dinophysis* and associated DSP toxicity may continue to plague this region in the near future.

### 3.3. DSTs in the USA

The first advance warning of a toxic *Dinophysis* bloom was facilitated by an Imaging FlowCytobot deployed in Port Aransas, Texas in 2008 [12]. Data from this video and flow cytometric monitoring system resulted in the closure of commercial oyster harvest due to concentrations of DSTs above the guidance level. During that event, only OA was found in oysters with over 98% in the 7-*O*-acyl ester fatty acid derivative form [13]. In contrast, in Washington State, mussels contain <50% of the fatty acid derivatives, while oysters have >50% and littleneck clams >80% of the fatty acid derivative forms (Table 1). In all shellfish tested from Washington State, DTX-1 was the primary toxin isomer. Similarly, on Salt Spring Island, British Columbia, Canada, samples collected at the time of the DSP event in 2011 showed DTX-1 and acyl DTX-1 in a 1:3 ratio [39]. Different shellfish species are known to metabolize the DSTs differently and the time of shellfish exposure to a *Dinophysis* bloom is a potential explanation for variable acylation of DSTs [40]. In contrast, on the U.S. east coast, both OA and DTX-1 were detected in mussel and clam from Massachusetts; whereas OA, DTX-1 and PTX-2 were found in water samples from Maryland [41]. These results show the wide range of DTXs in U.S. coastal waters, suggesting that either different *Dinophysis* species, different *Dinophysis* prey, varied environmental controls on toxin expression by *Dinophysis*, or a combination of these factors play a role in the diverse toxin composition. This suite of toxins necessitates chemical analysis by LC-MS/MS or by functional assay such as the protein phosphatase 2a test [42] for the quantitative assessment of total toxicity in shellfish samples. The presence of high proportions of acyl esters in shellfish demonstrates the need for sample hydrolysis as an essential step in the extraction procedure to avoid underestimation of DST concentrations.

### 3.4. Other Lipophilic Toxins

Azaspiracids are a relatively new class of lipophilic toxins comprising at least 30 congeners that were first detected in mussels (*Mytilus edulis*) from Ireland following a 1995 human outbreak of gastrointestinal illness and have since been detected in other bivalve species around the world, including oysters (*Crassostrea gigas*, *Ostrea edulis*), scallops (*Pecten maximus*), clams (*Tapes phillipinarum*), cockles (*Cardium edule*), and razor clams (*Ensis siliqua*) [43,44,45]. Cases of AZP and/or contaminated shellfish have since been documented in several other European countries [46,47,48,49], eastern Canada [50], and Morocco [51]. Of equal concern to the US is the importation of AZA contaminated shellfish. In 2008, contaminated Irish mussels caused a human AZP outbreak [52] resulting in the voluntary destruction of over 150 tons of shellfish by the industry [53]. Preliminary reports on AZAs suggest that they are highly toxic [44,50,54] with teratogenic potential to developing fish [55]. In three of the filtered plankton samples from Sequim Bay, AZA-2 was measured, pointing to the need to be alert for the presence of this toxin in shellfish. *Azadinium spinosum* is difficult to sample and identify by light microscopy due to its small size and has not yet been observed by SoundToxins or ORHAB partners, suggesting that other early warning methods, such as deployment of Solid Phase Adsorption Toxin Tracking (SPATT) samplers should be used to complement plankton and shellfish monitoring [56].

We now have clear evidence from the ORHAB and SoundToxins monitoring programs that DSTs, AZAs and YTX accumulate in Washington State shellfish, including oysters, mussels, varnish clams, geoduck clams, littleneck clams, and razor clams. Recent monitoring efforts in Washington State have confirmed the presence of species of the dinoflagellate genera *Prorocentrum*, *Dinophysis* and *Protoceratium*, all of which have been shown to be toxin producers in other regions of the world, such as Europe, New Zealand and Australia.

Further, the recent human illnesses in Puget Sound following consumption of well-cooked mussels have raised concerns regarding public health. The WDOH was notified of two suspected human DSP outbreaks in 2009 (April and August) and three confirmed illnesses in June 2011. In the 2011 cases, the illnesses were linked to shellfish, but there can be considerable potential for lipophilic toxin trophic transfer via other commercially significant species. To date, there have been no studies examining lipophilic toxin accumulation or trophic transfer in Puget Sound.

### 3.5. Lipophilic Toxin Regulation

The EU regulates the allowable levels of a variety of lipophilic shellfish toxins in product destined for market. European Union directives specify regulatory toxin levels of 160 μg OA equiv/kg (16 μg OA equiv/100 g) for DSTs and PTXs, 1 mg YTX equiv/kg (0.1 μg YTX equiv/100 g) and 160 μg AZA equiv/kg (16 μg AZA equiv/100 g) of shellfish meat (Regulation EC No 853/2004) [57]. In Canada, the guidance is that OA + DTXs must be <20 μg OA equiv/100 g shellfish; and in the US, the action level for total (esterified plus non-esterified) OA + DTXs is 16 μg/100 g shellfish established by the U.S. Food and Drug Administration (FDA) in 2011. Currently, the U.S. FDA has no guidance for PTXs and YTXs. The National Shellfish Sanitation Program (NSSP), a federal/state cooperative program recognized by the FDA and the Interstate Shellfish Sanitation Conference (ISSC) for the sanitary control of shellfish produced and sold for human consumption, currently have no guidance for appropriate testing methods for DSTs. However, Washington State has adopted the FDA guidelines for their routine monitoring of DSTs in shellfish for the protection of human health. The development or implementation of regulations in the US for new contaminants such as PTXs, YTXs and AZAs is not simple and straightforward. A detailed risk assessment would need to be formulated to determine if action should be taken, but first, a comprehensive analysis would be required encompassing data on the prevalence of toxic phytoplankton species, identification of factors influencing their growth and toxicity, in addition to an understanding of toxin persistence in the water column and food web, and information regarding toxicity. Such work will provide a critical first step in understanding the distribution of these toxins and the causative species, providing a framework with which to better understand and detect potential threats. Future research should also pay particular attention to chronic low-level exposures of humans and marine wildlife to these lipophilic toxins.

## 4. Experimental Section

### 4.1. Sample Collection

Surface seawater samples were collected each week by volunteer partners in the monitoring programs SoundToxins (Puget Sound) and ORHAB (outer Washington State coast). Personnel used a bucket for collections at the sampling sites either directly from the beach, or from piers or docks. A Niskin bottle, deployed to 2 m depth, was used to collect seawater from the Sequim Bay State Park pier. Whole water samples were then processed and analyzed for concentrations of DSTs and for *Dinophysis* cell counts from Sequim Bay, Discovery Bay and Quartermaster Harbor. At all other sites, *Dinophysis* cell relative abundance was estimated from net tow samples as absent, present, common, or bloom. Net tow samples for species identification were collected by hand using a 20-μm mesh phytoplankton net and preserved in 1% formalin.

Shellfish were collected from sentinel mussel cages maintained by the WDOH. Mussels were acclimatized for at least one week at the sentinel site before a sample was tested for biotoxins. In addition, the mussels that were used to restock cages were tested for biotoxins prior to sampling at the new sampling site. Source mussels came from locations that have a history of very rare to no biotoxin events. Shellfish testing was prioritized through collaboration with the SoundToxins and ORHAB phytoplankton monitoring programs (Figure 1B) [58,59]. When phytoplankton monitoring sites show a rapid increase in *Dinophysis* cell abundance from “absent” to “present or “present” to “common” or an abundance of >2000 cells/L, shellfish were given the highest priority for DST analysis by liquid chromatography tandem mass spectroscopy (LC-MS/MS). Clams and oysters were sampled at sites where subsistence harvests or commercial sales were planned.

### 4.2. Shellfish Preparation and Homogenization

Shellfish were rinsed with tap water then opened by cutting the adductor muscles. About 10–20 individual shellfish were pooled to make up at least 100 g of tissue per sample. Samples were drained for a few minutes to remove any excess water and then transferred to a glass blender for homogenization for 1 min. The resulting homogenates were stored in ultra-high performance polypropylene copolymer containers and frozen at −20 °C until analysis. The method for shellfish extraction was a modification of the EU-Harmonized Standard Operating Procedure for the Determination of Lipophilic Marine Biotoxins in Mollusks by LC-MS/MS [60]. An aliquot of sample homogenate (2.5 g) was accurately weighed into a 50 mL centrifuge tube (BD Falcon) and extracted with 12 mL of methanol by vortex mixing for 3 min. The mixture was centrifuged at 2500× *g* for 10 min and the supernatant transferred to a 25 mL volumetric flask. The residual pellet was re-extracted by homogenizing in 10 mL methanol for 1 min with a 10 mm stainless steel OmniProbe High Power Tissue Homogenizer [61] followed by centrifugation at 2500× *g* for 10 min. The supernatant was combined with the first extract and brought to 25 mL with methanol. The extract solution was mixed well and an aliquot was stored in an amber glass vial at −20 °C until analyzed by LC-MS/MS.

### 4.3. Filter Preparation and Extraction

Particulate (cellular) toxin samples were prepared by filtering 1 L of whole seawater using up to three filters (47 mm, 0.45 μm; Millipore, HAWP). Filters were folded in half with forceps, wrapped into aluminum foil packets and stored at −20 °C until analysis. The filter(s) for a single sample were placed into a 15 mL polypropylene centrifuge tube (BD Falcon), covered with 2 mL of 80% methanol, macerated by hand for about 3 min and bath sonicated for 60 min (Branson 5510 sonicator). Samples were then vortex mixed, centrifuged for 10 min at 2500× *g* and the supernatant solution was transferred to a glass vial. The residual pellets were re-extracted with 1 mL of 80% methanol by vortexing for 3 min followed by centrifugation for 10 min at 2500× *g*. The resulting supernatant solution was combined with the first extract and evaporated to below 2 mL with nitrogen at 25 °C. The final extract was brought to 2 mL with 100% methanol and filtered through a 0.22 μm PTFE syringe filter (Fisher Scientific) and stored at 4 °C in glass vials until analyzed by LC-MS/MS.

### 4.4. Tissue and Filter Extract Hydrolysis

The methanolic extract was hydrolyzed using a modification of the method published by Mountfort *et al*. [42]. A 125 μL aliquot of a 2.5 N NaOH solution was mixed with 1 mL of the extract and heated at 76 °C for 40 min in a tightly sealed 1.5 mL vial (Sun Brokers). The capped sample vial was weighed before and after heating to assure no evaporation. The hydrolyzed sample was neutralized with 125 μL of a 2.5 N HCL solution and filtered through a 0.22 μm PTFE syringe filter (Fisher Scientific). Non-hydrolyzed samples were filtered in the same way before analysis on the LC-MS/MS. Samples were stored at 4 °C until LC-MS/MS analysis on the same day as hydrolysis. Total free and esterified toxin levels were calculated with correction for the 25% volume increase from the additions of base and acid. All shellfish were hydrolyzed according to guidelines for their regulatory analysis, allowing for the quantification of both the acyl ester toxin forms (e.g., DTX-3) and the non-esterified toxin forms.

### 4.5. LC-MS/MS Analysis

The filtered shellfish and filter extracts were analyzed by UPLC (Acquity system, Waters Co., Milford, MA, USA) coupled with a triple quadrupole tandem mass spectrometer (MS/MS, ABSciex 5500, Framingham, MA, USA). For each sample, 10 μL of filtered extract was injected into the UPLC-MS/MS. The UPLC was equipped with a 0.2 μm pre-filter followed by a 2.1 × 4 mm C8 Security guard cartridge and a 2.1 × 100 mm, 5 μm Luna C8 reverse phase column (Phenomenex, Torrance, CA, USA) [62]. The acidic chromatographic conditions used in this study were described in the EU-Harmonised SOP [60] and by McCarron *et al*. [63]. The mobile phase was prepared daily from LC-MS grade solvents. The weak mobile phase (A) was 100% water with 2 mM ammonium formate with 50 mM formic acid and the strong mobile phase (B) was 95% acetonitrile and 5% water containing 2 mM ammonium formate and 50 mM formic acid. The column was maintained at 40 °C and a flow rate of 0.40 mL/min. The solvent program was a linear gradient starting at 70% A: 30% B to 10% A: 90% B over 8 min, then 10% A: 90% B for 4 min, a return to 70% A: 30% B for 0.5 min, and a constant 70% A: 30% B until the start of the next run. Total run time was 17 min.

Analytes were detected by alternating negative and positive ion mode using multiple reaction monitoring (MRM). The following negative transitions (precursor ion > product ion) were used for quantifying and confirming (confirming ions shown in parentheses) okadaic acid (OA) and dinophysistoxin-2 (DTX-2): *m/z* 803.5 > 255.0 (803.5 > 113.0); dinophysistoxin-1 (DTX-1): *m/z* 817.2 > 255.0 (817.2 > 113.0); yessotoxin (YTX): *m/z* 1141.5 > 1061.7 (1141.5 > 855.5). Positive ionization transitions were as follows, azaspiracid-1 (AZA-1): *m/z* 842.2 > 824.4 (842.2 > 806.3); azaspiracid-2 (AZA-2): *m/z* 856.2 > 838.3 (856.2 > 820.2); azaspiracid-3 (AZA-3): *m/z* 828.2 > 810.4 (828.2 > 792.5); pectenotoxin-2 (PTX-2): *m/z* 876.5 > 823.4 (876.5 > 213.2). The analytes were quantified with individual seven-point external calibration curves prepared in methanol from certified reference standards purchased from National Research Council Canada (Halifax, Nova Scotia). Standard calibration curve ranges were as follows: OA: 0.71 to 286 ng/mL; DTX-2: 0.44 to 177 ng/mL; DTX-1: 0.72 to 289 ng/mL; AZA-1: 0.03 to 12.4 ng/mL; AZA-2: 0.03 to 12.8 ng/mL; AZA-3: 0.043 to 17.3 ng/mL; PTX-2: 0.26 to 107.5 ng/mL; and YTX: 0.56 to 224 ng/mL. Each extract was run in the hydrolyzed and non-hydrolyzed form. Hydrolysis reduces esterified compounds to the parent form and allows the detection of total toxin for OA, DTX-2, DTX-1 and YTX but this process degrades PTX-2, AZA-1, AZA-2 and AZA-3 compounds therefore, these compounds were quantified from the non-hydrolyzed sample. A linear best fit was applied to each calibration curve (*R*^2^ > 0.99 in each case). Analyte concentration in tissue was calculated using peak area and adjusting for dilution factors, sample weight and toxin equivalent factor (TEF). The TEF for OA, DTX-1, PTX-2 and YTX was 1.0, and for AZA-2 was 1.8. Final reporting of the OA group was the sum of the adjusted concentrations of OA + DTX-1 and was reported as μg OA equiv/100 g tissue. DTX-3 was estimated as the difference between the hydrolyzed and non-hydrolyzed samples where a zero value was used for non-detects. Final reporting was in ng/L water for filter extracts. The limits of detection (LOD) and limits of quantitation (LOQ) in shellfish tissue for monitored toxins were, respectively: OA; 10 pg on column and 1.25 μg/100 g tissue, DTX-1; 10 pg on column and 1.25 μg/100 g tissue, DTX-2; 8 pg on column and 1.00 μg/100 g tissue, YTX; 10 pg on column and 1.25 μg/100 g tissue, PTX-2; 10 pg on column and 1.00 μg/100 g tissue, AZA-1; 1.3 pg on column and 0.13 μg/100 g tissue, AZA-2; 1.3 pg on column and 0.13 μg/100 g tissue, AZA-3; 2.0 pg on column and 0.20 μg/100 g tissue.

### 4.6. Dinophysis Cell Counts and Species Identification

Preserved whole water field samples were concentrated (10-fold) via settling and single 0.1 mL aliquots were counted for *Dinophysis* cells using a Palmer-Maloney counting chamber with a Zeiss Axiostar light microscope. *Dinophysis* cells were positively identified to the species level in selected preserved net-tow samples using published morphological characteristics [64] and verified by SEM. Sample preparation for SEM (JEOL 6360LV) imaging included dehydration in an ethanol series (30%–100%) with a final dehydration in hexamethyldisilazane (HMDS).

### 4.7. Fraser River Data

Fraser River flow data at the Hope, British Columbia station (08MF005) were obtained from the Environment Canada website [65]. Minimum, maximum, average, and 2011 flows were determined from the period of record (1912–2012) at this station. Data from 2012 were considered “provisional” when they were acquired from the Environment Canada website (November 2012).

## 5. Conclusions

Three cases of DSP in Sequim Bay in 2011, strongly illustrated the need to expand the analysis of shellfish for biotoxins in Washington State to include the DSTs. The monitoring of shellfish for DSTs in 2012 showed widespread occurrence of these toxins at concentrations above the guidance level in several species of shellfish, including blue mussel, California mussel, varnish clam, manila clam and Pacific oyster. Concentrations of total DSTs in blue mussels were at least twice those measured in other shellfish at the same site. The primary toxin isomer in shellfish and plankton samples was DTX-1 with *D. acuminata* as the primary *Dinophysis* species. Other lipophilic toxins in shellfish were PTX-2 and YTX with AZA-2 also measured in filtered phytoplankton samples. OA, AZA-1 and AZA-3 were all below the levels of detection by LC-MS/MS in Puget Sound shellfish. Shellfish from the Washington State outer coast showed low (<5%) levels of OA in their tissues including a sample from Ruby Beach that resulted in the first closure on the Washington State Pacific coast due to DSTs. The greater than average Fraser River flow during summer 2011 and 2012 may have provided an environment conducive to dinoflagellates and played a role in the prevalence of toxigenic *Dinophysis* in Puget Sound. Future work is needed to characterize the suite of environmental factors or “ecotypes” that are favorable for *Dinophysis* success and toxicity in Washington State in order to provide an early warning of these toxic algal blooms and to better understand why DSTs have become a greater problem in this region in recent years.
